# Surgical Excision of Sclerosing Mesenteritis, Exploration of an Unknown Mesenteric Mass

**DOI:** 10.7759/cureus.12546

**Published:** 2021-01-07

**Authors:** Thomas J Serena, Carolyn A Solomon Schnurr, John C Pui, Jeffrey R Gerken

**Affiliations:** 1 General Surgery/Vascular Surgery, Beaumont Health, Livonia, USA; 2 General Surgery, Michigan State University, Farmington Hills, USA; 3 Pathology, Beaumont Health, Farmington Hills, USA; 4 General Surgery, Beaumont Health, Farmington Hills, USA

**Keywords:** sclerosing mesenteritis, fat necrosis, mesenteric mass

## Abstract

Sclerosing mesenteritis is a rare and often benign condition characterized as a fibrotic disease consisting of non-suppurative inflammation of adipose tissue. Through mass effect, sclerosing mesenteritis can compromise the gastrointestinal lumen as well as mesenteric vessel integrity. There is a poor understanding of this disorder and its pathogenesis, which presents with various symptomatology and often without identification of inciting factors. Patients with sclerosing mesenteritis exhibit gastrointestinal and systemic manifestations including weight loss, fever, nausea, vomiting, diarrhea, and abdominal pain. This case presents a patient with a seven-month history of chronic, epigastric abdominal pain following laparoscopic surgery for acute uncomplicated appendicitis. The patient underwent work-up with computed tomography and magnetic resonance enterography that confirmed the presence of a mesenteric mass of unknown etiology located in the mid-epigastrium. Due to the inability to safely sample the mass, the patient underwent diagnostic laparoscopy, which was subsequently converted to an open procedure where excision of the mesenteric lesion was performed. Surgical pathology revealed fat necrosis with fibrosis, granulomatous inflammation, and dystrophic calcifications consistent with sclerosing mesenteritis. The patient was seen in follow-up with the resolution of her epigastric abdominal pain. This case report demonstrates a unique presentation of a symptomatic patient with a mesenteric mass not amenable to non-invasive biopsy. Complete excision of this lesser sac mass revealed sclerosis mesenteritis as the pathological cause.

## Introduction

Sclerosing mesenteritis is a rare and often benign condition characterized as a fibrotic disease consisting of non-suppurative inflammation of adipose tissue [1,2]. Through mass effect, sclerosing mesenteritis can compromise the gastrointestinal lumen and mesenteric vessel integrity [3-6]. The pathology has a diverse presentation and its pathogenesis is poorly understood. The process by which this occurs is thought to be non-neoplastic and non-suppurative.

This disease process correlates to a range of idiopathic chronic inflammatory diseases that occur within the intestinal mesentery causing significant fibrotic reactions [7]. A variety of nomenclature has been used to categorize the disease based on the degree of inflammation, fibrosis, and fat necrosis, with sclerosing mesenteritis being considered an umbrella term [7,8]. While most patients diagnosed with sclerosing mesenteritis are asymptomatic, documented cases have shown that patients can present with a variety of symptoms including abdominal distension, abdominal pain, weight loss, fever, nausea, and vomiting [9]. The disease itself is considered rare and tends to predominantly affect patients in their 50’s and 60’s [10]. Sclerosing mesenteritis is often found incidentally on radiographic imaging. It often is seen in the small bowel mesentery, secondary to accidental or surgical trauma to adipose tissue. Prior abdominal surgery, infection, autoimmune conditions, malignancy, and ischemia have all been theorized as etiologies [11]. 

This case was diagnosed as a result of clinical examination and imaging studies and then managed by the department of general surgery at a community-based surgical residency program. The case is unique as the imaging characteristics were more consistent with a mass of unknown origin and there was an inability to sample the lesion without operative intervention. Given the non-specific presentation of this condition, it is crucial to rule out other pathologies that can range from granulomatous and infectious diseases to benign or malignant masses [12]. This case is reported in accordance with SCARE criteria [13]. 

## Case presentation

A 55-year-old Caucasian female presented to the surgical office with a chief complaint of chronic epigastric abdominal pain for seven months duration. The patient was referred to the office after an emergency department visit for her chronic abdominal pain. The workup showed normal laboratory values with a nonspecific, lobulated soft tissue lesion located within the mesentery of the upper abdomen measuring up to 3.2 cm on computed tomography with intravenous and oral contrast. The patient noted her pain had developed and had been persistent since she underwent laparoscopic appendectomy for acute appendicitis roughly one year prior. She described her pain as sharp in nature with intermittent flares. The patient reported that eating exacerbated her symptoms. She endorsed anorexia as well as weight loss. Physical examination at that time was considered unremarkable. The patient’s medical history included gastroesophageal reflux disease, gastritis, depression, and a seizure disorder. The patient’s surgical history included a cholecystectomy for cholelithiasis, hemorrhoidectomy, tonsillectomy, and an appendectomy. Family and social history were non-contributory.

**Figure 1:**
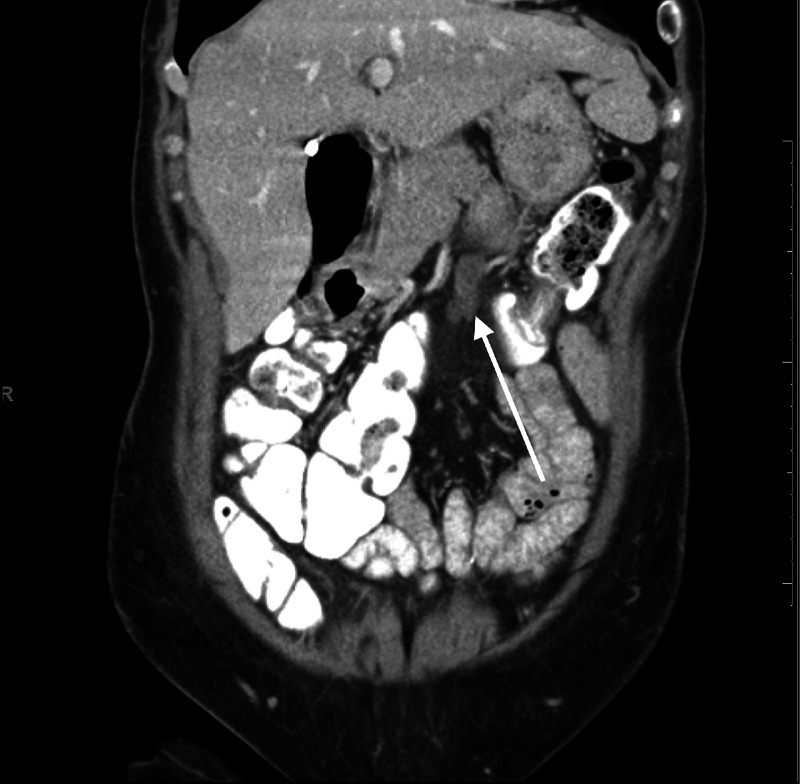
Diagnostic computed tomography Pre-intervention computed tomography of abdomen and pelvis with IV and oral contrast demonstrating a stable, previously reported 3.2 cm x 1.2 cm x 2.0 cm soft tissue mass of the anterior mesentery in the upper abdomen as demonstrated by arrow.

Based on the initial radiographic results and the location of the mesenteric lesion, the patient was referred for endoscopic evaluation and possible biopsy. They were then started on a trial of proton pump inhibitors. The patient had multiple visits to her primary care provider and was eventually transitioned to sucralfate as her symptoms were unresolved. The patient underwent esophagogastroduodenoscopy that revealed LA Grade B gastroesophageal reflux disease, a 2 cm hiatal hernia, and diffuse gastritis. Repeat computed tomography of the abdomen and pelvis with intravenous and oral contrast was performed revealing a lobulated lesion at the level of the kidneys within the anterior mesenteric fat, concerning for an enlarged lymph node or a neoplastic mass as demonstrated by the arrow in Figure 1. The mass was deemed inaccessible to endoscopic biopsy due to interference of surrounding structures and risks associated. 

**Figure 2:**
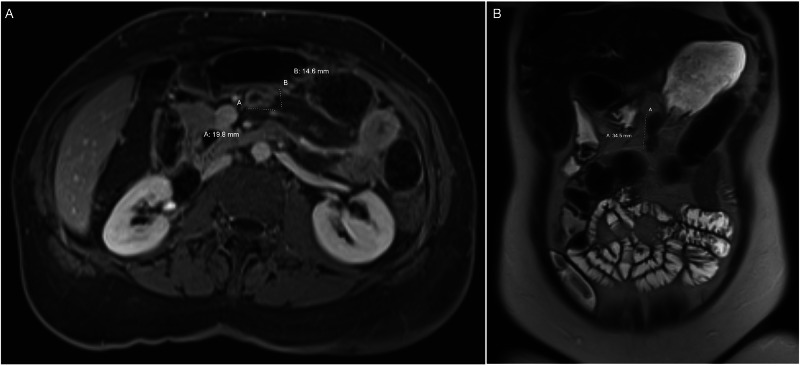
MRI enterography with and without gadolinium (A) Axial single slice and (B) coronal single slice showing a 2.0 x 1.5 x 3.5 cm transverse by anteroposterior by craniocaudal dimension ovoid enhancing lesion within the mesentery of the upper abdomen just posterior inferior to the gastric antrum.

The patient returned to the clinic for a follow-up examination. After an informed conversation regarding the possible etiologies of the mass and medical and surgical options, the decision was made to further characterize the soft tissue mass in preparation for surgery. Magnetic resonance enterography was performed which redemonstrated the previously reported mid-abdominal mesenteric mass located posterior-inferior from the gastric antrum, with etiology remaining unclear. In addition, there was a continued concern for an enlarged lymph node versus an underlying neoplastic process as seen in Figures 2A, 2B. Interventional radiology was consulted for further recommendations regarding potential image-guided biopsy of the mesenteric lesion. Due to the inability to safely perform a biopsy of the mass, surgical intervention was again considered. A discussion was held with the patient highlighting the persistent history of abdominal pain in the setting of an unknown mass. Pre-intervention informed consent centered on surgical approach, risks, and benefits of surgery. In reflection of the size, location of the mass, and maximization of surgical yield, the decision was made to attempt a diagnostic laparoscopy with possible wide local excision, possible laparotomy.

**Figure 3:**
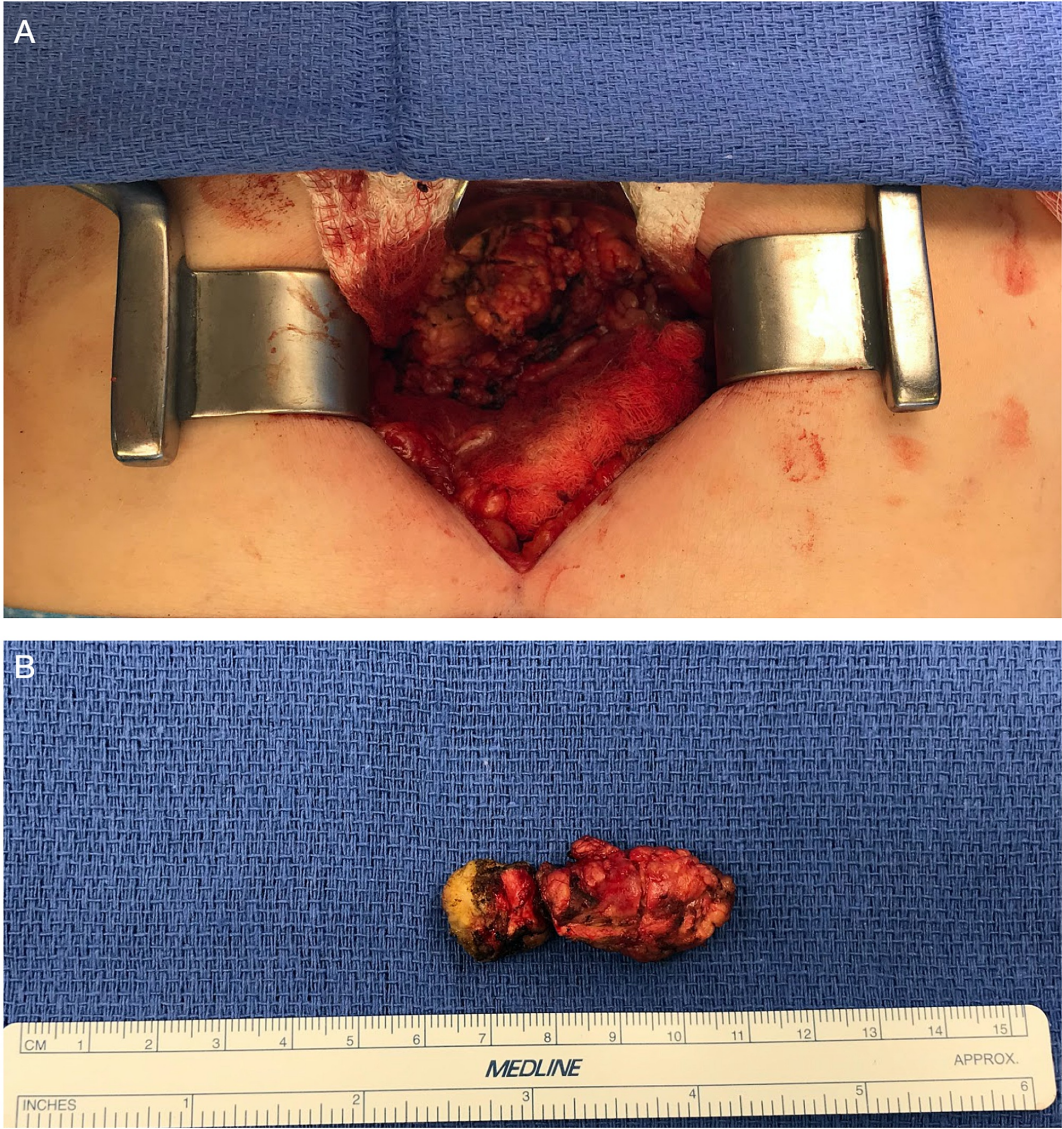
Intraoperative imaging Intraoperative images from mesenteric mass removal via a laparoscopic converted to open approach. (A) Shows open identification of the mass circumscribed. (B) Shows the mass status post surgical removal.

The patient was consented for and underwent diagnostic laparoscopic exploration. Upon entering the abdomen, the omentum, mesentery, and peritoneum were without signs of gross disease. Takedown of the gastrocolic ligament and omentum was performed and upon entering the lesser sac, there was a firm, mobile mass located inferior to the greater curvature of the stomach. Careful dissection was attempted via laparoscopic approach, but due to the dense nature of the mesentery and inability to properly characterize the location of the mass about the surrounding structures, the decision was made to convert to an open procedure. The mass was found to lay within the mesentery and was free of attachments to the underlying vasculature and pancreas. Careful sharp excision was performed to circumferentially inscribe the mass as demonstrated in Figure 3A. After meticulous dissection of the mass, it was safely removed in one piece as demonstrated in Figure 3B. The specimen was then sent to the department of pathology to further characterize the lesion. Due to the lack of gross neoplastic or desmoplastic reaction surrounding the mass, the decision was made to close primarily without further dissection. 

The postoperative period was uneventful and the patient remained stable throughout its entirety. The patient was discharged without complication on postoperative day two. The patient was seen in the office for follow-up at one week and at one-month post-operatively in which she endorsed the slow resolution of her previous symptoms. The authors can not say with certainty that her symptoms improved secondary to surgical intervention. 

**Figure 4:**
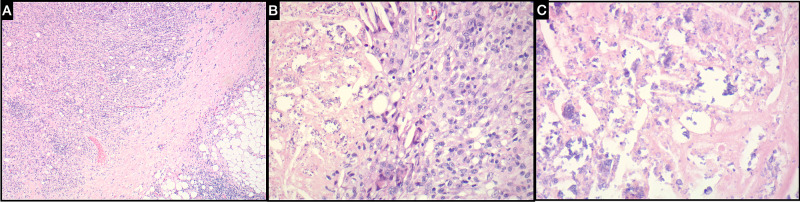
Histological examination of surgical specimen (A) Demonstrates omental adipose tissue composed of nodular inflammatory cells composed predominantly of histiocytes with interspersed lymphocytes (hematoxylin and eosin, original magnification 40×). (B) Showing histiocytes with foamy cytoplasm and surrounding areas of fat necrosis (hematoxylin and eosin, original magnification 200×). (C) Revealing granular basophilic particles representing dystrophic calcification dispersed throughout the areas of fat necrosis (hematoxylin and eosin, original magnification 400×).

Histological evaluation of the surgical specimens revealed adipose tissue with a nodular collection of inflammatory cells composed predominantly of histiocytes with interspersed lymphocytes. The histiocytes contained foamy cytoplasm and surrounding areas of confluent fat necrosis with associated cholesterol clefts. Granular basophilic particles representing dystrophic calcification were dispersed throughout the areas of fat necrosis. Examination by polarized light microscopy did not show foreign material. Special stains for infectious organisms were negative. Histological examination of the surgical specimen is seen in Figures 4A, 4B, 4C. The pathological evaluation concluded the lesion in question was mesenteric fat necrosis without any evidence of atypia or malignancy.

## Discussion

Sclerosing mesenteritis was first described in 1924 as a retractile mesenteritis [14]. Since then, it has adopted many terms such as mesenteric panniculitis, mesenteric fibrosis, mesenteric lipodystrophy, liposclerotic mesenteritis, etc. This pathology represents a spectrum of fat necrosis secondary to chronic inflammation leading to a fibrotic reaction. While benign in nature, this entity ranges from simple sclerosis to retraction of the mesentery and possible encasement of bowel and vasculature [15]. The disease process itself is very rare. A prospective analysis, which reviewed 7620 consecutive abdominal CT scans, was performed to assess the overall prevalence of sclerosing mesenteritis. The study identified and confirmed the disease histologically presents in roughly 0.6% of the total imaging analyzed with female predominance [16]. 

The etiology of this disease process is unknown in many cases. There have been several theories proposed, that span from ischemia, trauma, or secondary to a neoplastic process, most commonly non-Hodgkin's lymphoma. In addition, it often presents in three different forms: type I presenting with diffuse mesenteric thickening (42%), type II presenting with a single discrete mass (32%), and type III with multiple discrete masses (26%) [17].

The treatment for sclerosing mesenteritis is often observed as the disease is most commonly self-limiting. If the disease is severe or has a protracted course, first-line therapy is a combination of prednisone and tamoxifen. Other multifactorial treatments have been proposed, however, most have limited power. Current prospective trials testing the efficacy of thalidomide are underway. Other case reports present success with anti-inflammatory medications [18].

Most patients with sclerosing mesenteritis present with non-specific abdominal pain. Oftentimes, a diagnosis of sclerosing mesenteritis can be achieved through imaging alone as it has relatively specific findings of well-demarcated mesenteric mass-like lesions with hazy surrounding soft-tissue attenuation on computed tomography. When imaging characteristics are not diagnostic and the clinical picture is discordant, sampling of the tissue is recommended [19]. In a review of the clinical spectrum of disease, surgery is seldom offered except for cases of persistent symptomatology or patients with obstruction. Furthermore, there is concern that given this is a chronic inflammatory reaction, an R0 resection is not feasible. This case presents a rare circumstance where imaging cannot rule out a malignant process, and minimally invasive biopsy was not attainable. 

The diagnostic approach for the case presented was utilized as a step-up fashion to surgery. In exploring the peritoneum via laparoscopy and subsequent laparoscopic dissection into the lesser sac, concern for metastatic involvement was reduced. Complete resection was performed once the mass was noted to be free from surrounding structures in an attempt to remove pathological tissue and increase surgical yield. An alternative approach to consider could have been laparoscopic core-needle biopsy. Excisional biopsy was favored, however, given the extent of mesenteric involvement, relation to surrounding vasculature, and proximity to vital organs. 

## Conclusions

Sclerosing mesenteritis is a benign and rare condition characterized by non-suppurative inflammation of adipose tissue. As a spectrum of disease processes, its presentation, and the pathological impact can vary. This case presents a patient with a mesenteric mass in the setting of a seven-month history of chronic epigastric abdominal pain following laparoscopic surgery for acute uncomplicated appendicitis. After a non-diagnostic work-up and inability to obtain a tissue sample safely, the patient underwent diagnostic laparoscopy with conversion to open and subsequent complete excision. Surgical pathology demonstrated fat necrosis with fibrosis, granulomatous inflammation, and dystrophic calcifications without evidence of atypia or malignancy. This case demonstrates a unique approach to an unknown mesenteric mass found to be consistent with sclerosing mesenteritis.
